# The accumulation profiles of terpene metabolites in three Muscat table grape cultivars through HS-SPME-GCMS

**DOI:** 10.1038/s41597-019-0321-1

**Published:** 2020-01-02

**Authors:** Lei Sun, Baoqing Zhu, Xuanyin Zhang, Huiling Wang, Ailing Yan, Guojun Zhang, Xiaoyue Wang, Haiying Xu

**Affiliations:** 1 Beijing Academy of Forestry and Pomology Sciences, Beijing, 100093 China; 2 Beijing Forestry University, Beijing, 100083 China; 3 Key Laboratory of Biology and Genetic Improvement of Horticultural Crops (North China), Ministry of Agriculture and Rural Affairs, Beijing, 100093 China; 4 Beijing Engineering Research Centre for Deciduous Fruit Trees, Beijing, 100093 China

**Keywords:** Plant breeding, Plant sciences

## Abstract

Aroma is an important parameter for table grapes and wines; terpenes are typical compounds in Muscat-type grape cultivars and can be easily perceived by humans because of their low olfactory threshold. Volatile terpenes contribute directly to the aroma character, while glycoside-bound terpenes are potential aromatic compounds and can be changed to their volatile forms via hydrolysis. With gas chromatography-mass spectrometry and a solid-phase microextraction method, an automatic data analysis platform was constructed; terpene compounds were identified and quantified from three table grape cultivars at three stages during berry development, and the raw data were deposited in MetaboLights. Terpene metabolite accumulation profiles are presented in this article for integrative analysis with the transcriptome data and phenotypic data to elucidate the important candidate genes and mechanism for terpene biosynthesis. Our method has applications in the identification and quantification of terpene compounds with very low or trace concentrations.

## Abstract (summary)

**Table:** 

Measurement(s)	Terpene Compound
Technology Type(s)	gas chromatography-mass spectrometry • solid-phase micro-extraction
Factor Type(s)	cultivar • developmental stage • biological replicate
Sample Characteristic - Organism	Vitis vinifera

Machine-accessible metadata file describing the reported data: 10.6084/m9.figshare.10327715

## Background & Summary

The aroma character of Muscat grape cultivars has been thoroughly studied; monoterpenes are the main compounds in Muscat grapes, in the form of terpene alcohols, alkenes, aldehydes, and their oxides. The dominant terpenes in grapes are linalool, geraniol, nerol, terpineol and citronellol. Terpenes exist in two forms: the free form is volatile and directly contributes to flavor, while the glycoside form is non-volatile but can be transformed into the volatile form via hydrolysis^1–6^. Generally, bound terpenes are more abundant than the free form^7^. In grapes, terpenes mainly exist in the vacuoles of pericarp cells and in the flesh in some varieties^8^; their contents are affected by the genotype^9–11^, development stage^12,13^, environment and management^14–17^.

Numerous methods have been used for the detection and analysis of aromatic compounds. The extraction strategies mainly include liquid-liquid extraction, steam distillation, solid-phase extraction, supercritical fluid extraction, and static headspace extraction^18–21^. Among these, solid-phase microextraction (SPME) has been widely used for the extraction of volatile compounds since 1996^22^. SPME has several advantages over other sample preparation techniques: no organic solvents are needed, the process is easily automated, and small sample sizes are required^23^. The target compounds are not compromised, and it is easy to set up the automation system and connect to downstream analytical instruments^24,25^. These features make SPME particularly well suited for studies of a large number of samples. However, the selection of fiber type and model is complicated in the SPME system and should be determined by the extracted compounds and preliminary experiments; additionally, SPME is not applicable when the compounds are not easily volatilized.

Data analysis is a critical step when handling large amounts of metabolic data, and efficient software for peak identification and deconvolution should be optimized to obtain as much information as possible from a sample^26,27^. Fragment ions from isomers are highly overlapped between different compounds, and this coelution is one of the difficulties in separating compounds, especially for complicated samples. The automated mass spectral deconvolution and identification system (AMDIS) was developed by the American National Standards Institute and is very powerful when dealing with background noise, single peak extraction and shift correction; the detection limit can reach 10^−12^ g/l^28^.

In this study, an automatic data analysis platform was constructed, and the contents of the free and bound forms of terpenes were analyzed in 27 table grape samples. Terpene compounds were identified by comparing their retention indices and mass spectra with reference standards and the NIST11 library. Quantification was carried out with standard curves. A total of 28 terpenes were identified. Regarding their structure and category, these terpenes included 5 cyclic alkenes: β-myrcene, (Z)-β-ocimene, (E)-β-ocimene, (E,Z)-allo-ocimene, and (Z)-allo-ocimene; 6 cyclic alcohols: nerol, geraniol, β-citronellol, linalool, γ-geraniol, and isogeraniol; 3 cyclic aldehydes: geranial, neral, and citronellal; 6 cyclic ethers: nerol oxide, cis-rose oxide, trans-rose oxide, cis-furan linalool oxide, trans-furan linalool oxide, and cis-pyran linalool oxide; 1 cyclic acid: geranic acid; 3 acyclic alkenes: limonene, γ-terpinene, and terpinolene; and 2 acyclic alcohols: α-terpineol and 4-terpineol.

The contents of these terpenes could be used for correlation analysis with transcriptome data. The data will be useful for elucidating the mechanism of terpene biosynthesis in table grapes and will provide information for future breeding.

## Methods

### Overview of the experimental design

**Fig. 1:**
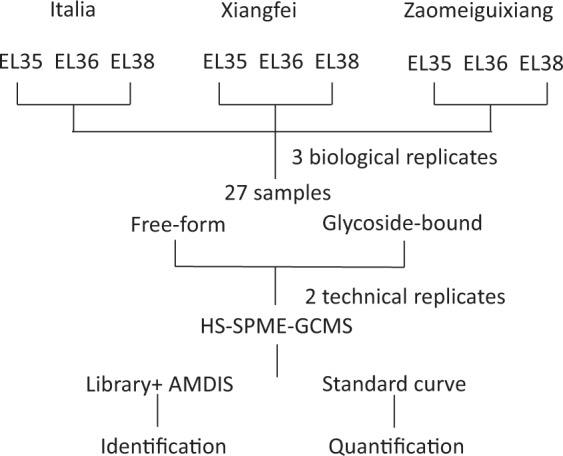
flowchart of the experimental design.

The berries of three genotypes were collected at three developmental stages. Approximately 300 grape berries were randomly collected for each replicate, and three replicates were harvested for each stage. The experimental design and analysis pipeline are shown in Fig. 1.

## Materials and Methods

### Sample collection

Three *Vitis vinifera* cultivars were used for volatile metabolite analysis: ‘Xiangfei’ was registered by our team in 2000 and has a strong Muscat flavor with a green to golden skin color, ‘Italia’ is a famous mid-late-season table grape cultivar originated in Italy and has moderate Muscat flavor, and ‘Zaomeiguixiang’ has a purple-reddish color and a strong Muscat flavor.

The vines were planted in the experimental field at the Beijing Academy of Forestry and Pomology Sciences in China (39°58′N and 116°13′E) under a plastic cover and trained into a two-wire vertical trellis system with a 2.5-m row space and a 0.75-m plant space. Berry samples from three vines in 2017 were harvested at the development stages corresponding to EL35, EL36, and EL38^29^. The berry begins to color and soften at EL 35, the completely colored version with intermediate Brix occurs at EL 36, and the berry reaches harvest ripeness at EL38. At each stage, three replicates were harvested; for each replicate, approximately 300 grape berries were randomly collected from ten vines. The berries were frozen in liquid nitrogen and stored at −80 °C.

### Extraction of volatile compounds

The extraction of volatile compounds from the grape berries followed Wen’s published method with minor modifications^30^. The grape berries (approximately 100 g), after discarding seeds and pedicels, were ground and then blended with 1 g of polyvinylpolypyrrolidone (PVPP) under liquid nitrogen. The resultant mixture was macerated at 4 °C for 4 hours and then centrifuged at 8,000 rpm for 15 min at 4 °C to obtain a clear juice. Five milliliters of the liquid were mixed with 1 g of sodium chloride and 10 µL of 1.00808 g/L 4-methyl-2-pentanol in a 20 mL vial (Agilent, 5188-2753 Santa Clara, California, United States) capped with a silicone septum (Agilent, 8010-0139). Two technical replicates were performed for each sample.

### Extraction of glycoside-bound compounds

Ten milliliters of methanol and 10 milliliters of water were added in advance to the Cleanert PEP-SPE resins (Bonna-agela Technologies, China). Two milliliters of the clear juice were passed through the Cleanert PEP-SPE column. Water-soluble compounds were eluted with 2 mL of water, free volatiles were washed out with 10 mL of dichloromethane, and then the bound terpenes were eluted with 20 mL of methanol and collected in a round flask. The flow rate was approximately 2 mL/min. The methanol eluate was concentrated to dryness by a rotary evaporator under a vacuum at 30 degrees and then redissolved in 10 mL of a 2 M citrate-phosphate buffer solution (pH 5.0). Subsequently, 4.9 mL of the solution was transferred to a tube, and 100 μL of AR 2000 was added for incubation at 40 degrees for 16 hours. Afterwards, the liquid (5 mL) was mixed with 1 g sodium chloride and 10 µL of 1.00808 g/L 4-methyl-2-pentanol in a 15-mL vial capped with a PTFE-silicone septum.

### GC-MS conditions

The volatile compounds were absorbed using headspace solid-phase microextraction (HS-SPME) and then analyzed using an Agilent 7890B-5977A gas chromatograph-mass spectrometer (GC-MS). The autosampler (CTC-PAL RSI85, CTC Analytics, Zwingen, Switzerland) was operated in SPME mode with an SPME fiber (57348-U, Supelco, Bellefonte, PA, USA). The sample vial was initially incubated at 40 °C for 30 min under agitation at 250 rpm, and then the preconditioned SPME fiber was inserted into the headspace of the vial to extract volatiles for 30 min at 40 °C under the same agitation conditions. Then, the SPME fiber was immediately inserted into the GC injection port at 250 °C for 8 min to desorb the volatiles. A 60 m × 0.25 mm HP-INNOWAX capillary column with a 0.25 μm film thickness (J&W Scientific, Folsom, CA, USA) was used to separate the volatile compounds under a 1 mL/min flow rate of helium (carrier gas). The oven temperature program was set as follows: 50 °C for 1 min, increase to 220 °C at 3 °C/min and hold at 220 °C for 5 min. The ion source was maintained at 250 °C with an MSD transfer line temperature of 250 °C. Mass scans were performed from m/z 30-350 with an ionization voltage of 70 eV.

### Identification of the compounds

The identification of volatile compounds was based on the retention index of reference standards and mass spectra matching using the standard NIST 11 library. A comparison of the retention index with those reported in the literature was used when standards were not available. Automated mass spectral deconvolution and identification system software (AMDIS, version 2.69, NIST, Washington, DC, USA) was used for peak deconvolution. The parameters for deconvolution were set as follows: component width = 20; adjacent peak subtraction = 2; resolution = high; sensitivity = low; and shape requirements = medium. A self-built library was used to search and match target compounds. Parameters for peak detection were set with default values. The identification information is listed in Table 1.

**Table 1:** Identification information of the compounds.

Compounds	Retention time	Retention Index	Molecular formula	Standard for quantification	Fragmentation	Ion for identification	Calibration Curves
β-Myrcene	13.977	1173	C_10_H_16_	β-Myrcene	41/69/93	41/93	y = 3350.8x − 1.0946
Phellandrene	13.756	1165	C_10_H_16_	Limonene	77/93/91/136	77/93	y = 306.8x + 0.9404
β-trans-Ocimene	16.662	1251	C_10_H_16_	β-Myrcene	79/93/121	79/93	y = 3350.8x − 1.0946
γ-Terpinene	17.184	1253	C_10_H_16_	Terpinolene	91/93/121/136	93/121	y = 300.19x + 1.3797
β-cis-Ocimene	17.386	1242	C_10_H_16_	β-Myrcene	79/93/121	79/93	y = 3350.8x − 1.0946
Terpinolene	18.747	1291	C_10_H_16_	Terpinolene	91/93/121/136	93/121	y = 300.19x + 1.3797
Cis Rose oxide	21.629	1338	C_10_H_18_O	Rose oxide	69/139/154	139	y = 37.312x + 3.259
trans-Rose oxide	22.289	1375	C_10_H_18_O	Rose oxide	69/139/154	139	y = 37.312x + 3.259
(E,Z)-Allo-Ocimene	22.518	1381	C_10_H_16_	β-Myrcene	105/121/136	105/121	y = 3350.8x − 1.0946
Allo-Ocimene	23.471	1397	C_10_H_16_	β-Myrcene	105/121/136	121/136	y = 3350.8x − 1.0946
nerol oxide	26.414	1480	C_10_H_16_O	Nerol	41/67/68/83	68/83	y = 930.79x + 8.9527
Citronellal	26.776	1765	C_10_H_18_O	Citronellal	41/69/95/121	41/69	y = 70680x + 43.97
Neral	34.916	1755	C_10_H_16_O	citral	41/69/109	41/69	y = 52.961x + 2.6933
geranial	37.006	1680	C_10_H_16_O	citral	41/69/84	41/69	y = 52.961x + 2.6933
cis-isogeraniol	39.415	1818	C_10_H_18_O	Geraniol	41/81/109/121	41/81/109	y = 1476.8x + 0.392
Geranic acid	56.744	2340	C_10_H_16_O_2_	Geranic acid	41/69/100	41/69/100	y = 6818.4x + 7.8939
Linalool	29.627	1581	C_10_H_18_O	Linalool	55/71/93/121	93	y = 396.8x + 3.2904
α-Terpineol	35.647	1710	C_10_H_18_O	α-Terpineol	59/93/121/136	136	y = 958.5x + 5.9227
Nerol	39.384	1797	C_10_H_18_O	Nerol	41/69/93	69	y = 930.79x + 8.9527
Geraniol	41.043	1857	C_10_H_18_O	Geraniol	41/69	69	y = 1476.8x + 0.392
Citronellol	37.966	1766	C_10_H_20_O	β-Citronellol	41/69/82/95	69	y = 1323.9x + 0.3414
Limonene	15.223	1205	C_10_H_18_O_2_	Limonene	68/93/121/136	68/93	y = 306.8x + 0.9404
cis-furan linalool oxide	25.175	1448	C_10_H_18_O_2_	Linalool	41/43/59/68	59/94	y = 396.8x + 3.2904
trans-furan linalool oxide	26.567	1477	C_10_H_18_O_2_	Linalool	41/43/59/68	59/94	y = 396.8x + 3.2904
Linalool oxide pyranoside	37.143	1691	C_10_H_18_O_2_	Linalool	41/43/59/68	68	y = 396.8x + 3.2904
trans-isogeraniol	39.751	1829	C_10_H_18_O	Geraniol	41/81/109/121	121	y = 1476.8x + 0.392

### Quantification of the compounds

The quantification procedure used in this study was based on previous work by Cai^31^. A synthetic matrix was prepared in distilled water containing 7 g/l tartaric acid and 200 g/l glucose. The pH was adjusted to 3.3 with a 5 M NaOH solution. The standard solutions contained thirteen terpenes and had fifteen levels, each of which was the halve concentration of the previous level. These solutions were extracted and analyzed under the same conditions as those of the grape samples. An internal standard (4-methyl-2-pentanol, 10 mL, 1.0018 g/l in water) was added to both the standard solutions and samples before aroma extraction and analysis. The calibration curves for analytes were established with regression coefficients above 98%. The concentrations of volatile compounds for which it was not possible to establish calibration curves were estimated on the basis of the equations of compounds with the same functional groups and/or similar numbers of C atoms. Data management and analysis were performed using ChemStation software (Agilent Technologies, Santa Clara, California, United States).

## Data Records

A total of 108 raw metabolite data files were deposited in MetaboLights^32^, including the free-form and glycoside-bound data of 27 samples (two technical replicates). The relative peak areas, the concentration of all samples and the statistically analyzed results were deposited in figshare^33^.

## Technical Validation

There were three biological replicates and two technical replicates for each sample. The final concentrations of the free and bound forms of the compounds were analyzed by statistics and are listed in Online-only Table 1 and Online-only Table 2.

## Usage Notes

**Fig. 2:**
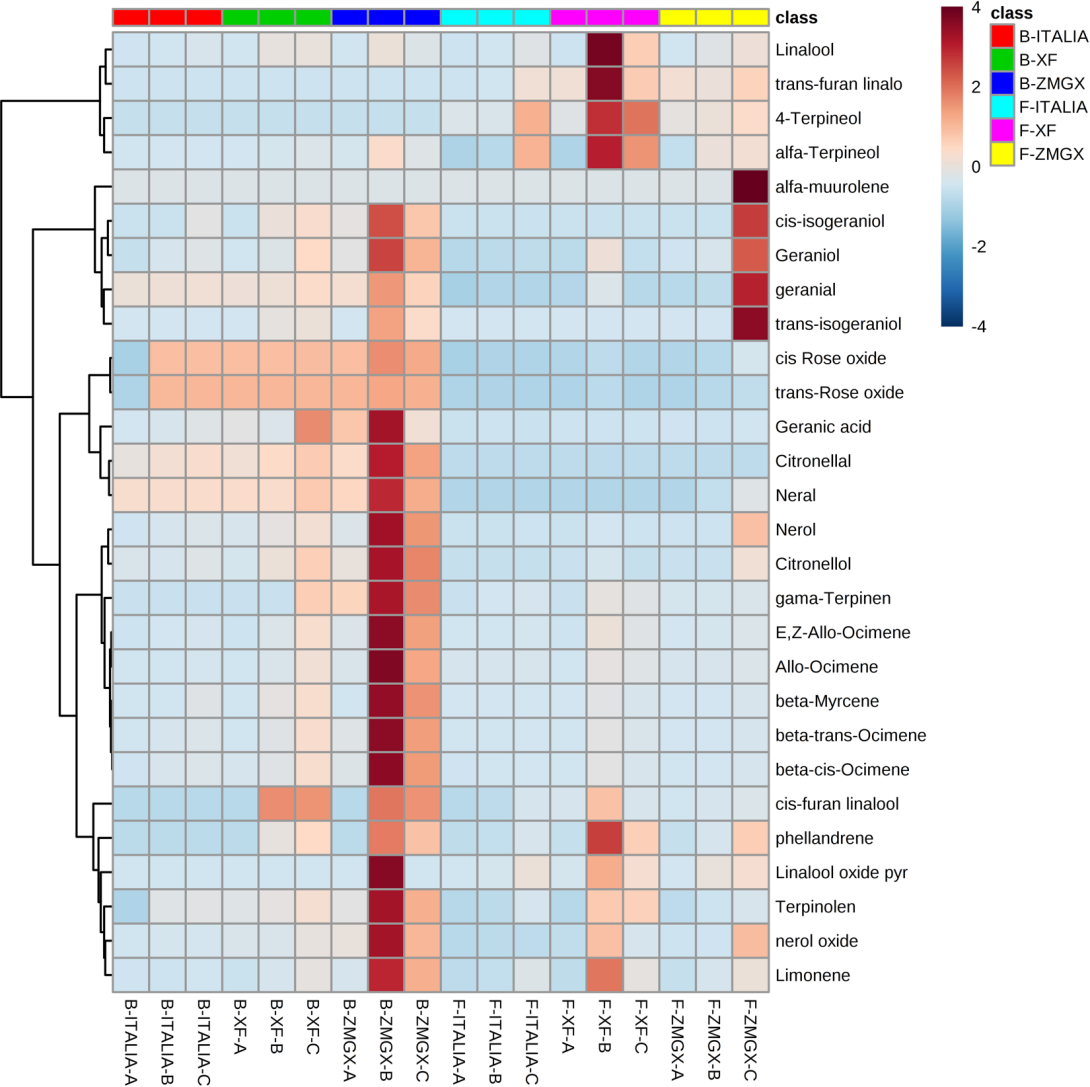
Heatmap of terpene concentration in table grapes during three developmental stages.

**Fig. 3:**
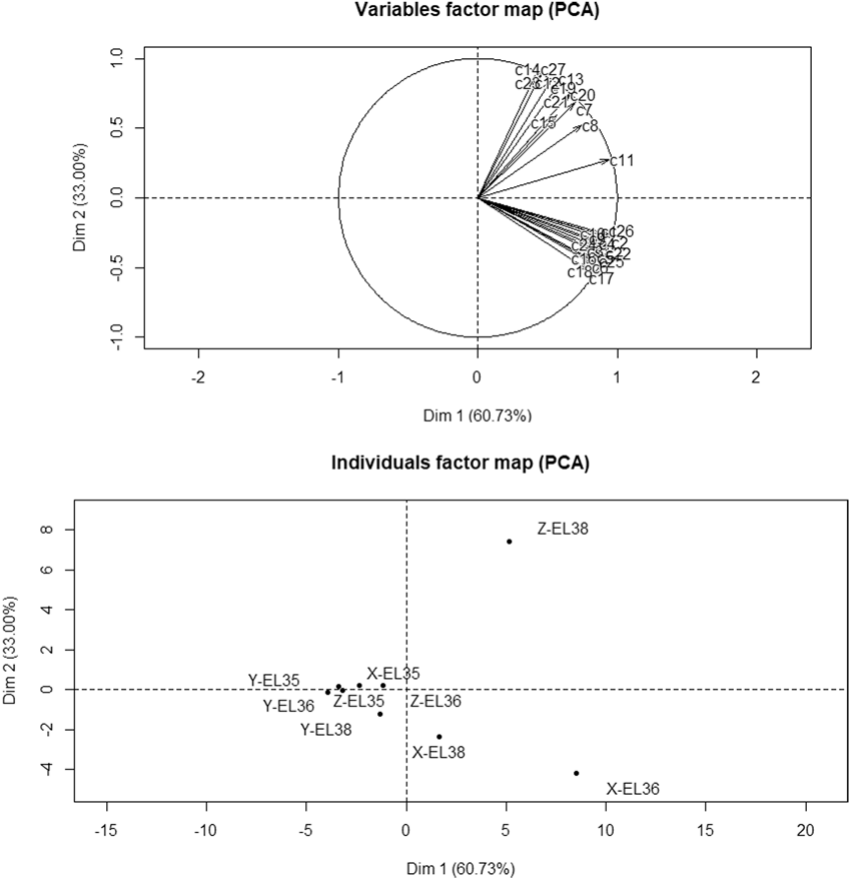
PCA analysis of terpene concentration at three stages.

The final concentration of all the samples were presented in excel files and deposited in the figshare. Figure 2 shows the heatmap of terpene concentration for three cultivars at three stages. Figure 3 shows the PCA analysis results.

The B represents the glycoside-bound form. F represents the free volatile form. The A, B, and C at the bottom of the X axis represent the berry developing stages EL35, EL36 and EL38, respectively. XF and ZMGX represent the cultivars Xiangfei and Zaomeiguixiang, respectively.

X stands for Xiangfei, Y stands for Italia, Z stands for Zaomeiguixiang, the terpene names of C1-C26 were shown in Table 1.

### Supplementary information

Supplementary File 1

## Data Availability

The code for the heatmap and PCA analysis is available in Supplementary file 1.

## Online-only Tables

**Online-only Table 1:** The concentration of free-form terpenes.

terpenes	X						Y						Z					
	EL35	SE	EL36	SE	EL38	SE	EL35	SE	EL36	SE	EL38	SE	EL35	SE	EL36	SE	EL38	SE
β-Myrcene	5.55c	0.52	181.88a	16.93	66.79b	8.82	3.18c	0.12	6.32c	3.57	19.67c	6.92	7.73c	1.56	20.08c	14.50	70.36b	12.11
phellandrene	1.4c	0.20	25.85a	2.12	10.73b	1.33	0.59c	0.03	1.19c	0.61	3.53c	1.02	1.26c	0.22	3.31c	1.83	10.94b	2.07
β-trans-Ocimene	nd	N/A	52.92a	3.02	22.37b	4.30	3.41ef	0.06	3.37ef	1.06	7.51d	1.85	3.91def	0.33	6.93de	3.29	16.73c	2.43
γ-Terpinene	nd	N/A	3.9a	0.62	2.93b	0.38	nd	N/A	1.24e	0.14	1.69cd	0.23	1.35de	0.02	1.42de	0.17	1.97c	0.17
β-cis-Ocimene	3.55ef	0.17	105.61a	6.19	46.92b	8.81	nd	N/A	4.56ef	2.31	13.54d	4.24	5.54def	0.68	11.94de	6.85	32.81c	5.10
Terpinolene	1.29c	0.02	16.65a	0.94	15.34a	7.17	1.13c	0.01	1.8c	0.96	5.97b	1.34	2.11c	0.09	4.28b	0.83	6.16b	0.68
Cis-Rose oxide	1.17d	0.03	2.45b	0.10	1.2d	0.03	nd	N/A	1.01d	0.02	1.06d	0.03	1.15d	0.04	1.98c	0.39	5.48a	0.72
trans-Rose oxide	nd	0.00	1.31b	0.03	nd	0.00	nd	N/A	nd	N/A	nd	N/A	nd	0.01	1.16c	0.11	2.17a	0.23
(E,Z)-Allo-Ocimene	nd	N/A	28.99a	1.97	17.03b	2.05	2.55ef	0.02	3.1de	0.73	5.9d	1.34	3.48de	0.22	5.34de	2.17	12.04c	1.56
Allo-Ocimene	nd	N/A	11.51a	0.60	7.58b	0.69	2.54e	0.02	2.74de	0.24	3.7d	0.44	2.83de	0.08	3.46de	0.71	5.94c	0.57
nerol oxide	23.81cde	4.04	202.76a	13.86	60.58b	7.13	8.1e	0.66	12.83e	1.21	16.56de	1.05	39.42bce	3.81	44.86bc	11.02	208.69a	25.86
Neral	nd	N/A	nd	N/A	nd	N/A	nd	N/A	nd	N/A	nd	N/A	nd	0.07	2.71b	0.55	7.83a	1.05
geranial	2.27ab	0.18	9.38b	0.53	2.57ab	1.59	nd	N/A	1.83ab	0.11	2.03ab	0.57	3.07ab	1.20	4.22ab	6.43	49.18a	6.32
cis-isogeraniol	nd	N/A	nd	N/A	nd	N/A	nd	N/A	nd	N/A	nd	N/A	nd	N/A	nd	N/A	43.33a	7.20
Geranic acid	42.73cd	3.44	67.02bcd	5.26	75.23bc	25.84	13.7d	3.01	40.9cd	1.81	32.04cd	8.94	102.6ab	30.68	40.85cd	36.64	144.59a	54.50
Linalool	8.43e	1.63	1473.24a	85.83	407.39b	50.65	3.87e	0.19	26.27e	22.51	118.76d	35.07	23.33e	1.93	118.88d	43.87	213.87c	15.84
4-Terpineol	1.32e	0.27	9.52a	0.36	7.11b	0.81	1.07e	0.09	1.05e	0.65	4.96c	0.74	1.7de	0.10	2.04de	0.32	2.92d	0.24
α-Terpineol	10.7e	0.62	181.57a	11.42	118.04b	16.39	9.05e	0.21	16.85e	13.66	99.24c	13.21	23.63e	1.16	53.25d	5.86	59.13d	6.90
Nerol	17.76b	1.78	149.16b	9.59	33.62b	15.91	2.7b	1.00	16.12b	2.82	26.58b	5.88	23.52b	10.26	43.46b	76.11	1572.38a	283.38
Geraniol	49.91d	5.06	335.44b	24.83	90.86cd	67.12	25.84d	5.20	45.67d	6.52	65.83d	19.63	137.19cd	60.46	187.85c	369.05	1035.58a	387.88
Citronellol	3.86c	0.54	13.63b	0.90	2.02c	1.27	1.8c	0.05	2.03c	0.06	1.94c	0.18	3.6c	0.87	3.94c	5.18	40.86a	6.09
Limonene	2.08e	0.22	82.01a	4.38	21.67bc	6.38	1.13e	0.06	3.69e	3.08	16.07cd	4.46	4.83e	0.40	12.54d	4.33	25.64b	3.89
α-muurolene	nd	N/A	nd	N/A	nd	N/A	nd	N/A	nd	N/A	nd	N/A	nd	N/A	nd	N/A	3.09a	0.09
cis-furan linalool oxide	3.35bc	0.34	12.86a	0.60	3.71b	0.15	nd	0.00	0.5d	0.54	3.44bc	0.17	2.8c	0.13	3.43bc	0.33	4.05b	0.15
trans-furan linalool oxide	1.73c	0.31	10.02a	0.46	3b	0.24	nd	0.00	0.25d	0.25	1.74c	0.24	1.85c	0.15	1.44c	0.25	2.7b	0.18
Linalool oxide pyranoside	0.45d	0.20	8.12a	0.44	3.68b	0.28	nd	N/A	0.42d	0.44	2.79c	0.28	0.27d	0.06	2.51c	0.23	3.65b	0.13
trans-isogeraniol	nd	N/A	nd	N/A	nd	N/A	nd	N/A	nd	N/A	nd	N/A	nd	N/A	nd	N/A	39.26a	6.24

X, Y, and Z represent the cultivars Xiangfei, Italia and Zaomeiguixiang, respectively. Different letters represent significance levels of P < 0.05, and nd stands for nondetected. N/A stands for not available and SE stands for standard error.

**Online-only Table 2:** The concentration of the bound-form terpene.

terpenes	X						Y						Z					
	EL35	SE	EL36	SE	EL38	SE	EL35	SE	EL36	SE	EL38	SE	EL35	SE	EL36	SE	EL38	SE
β-Myrcene	nd	N/A	226.64cd	2.85	463.98c	12.03	nd	N/A	nd	1.62	168.21cd	6.48	nd	N/A	2465.33a	79.12	1262.42b	26.56
phellandrene	nd	N/A	5.79c	0.01	9.51bc	0.33	nd	N/A	nd	N/A	nd	N/A	nd	N/A	20.10a	0.50	12.42b	0.15
β-trans-Ocimene	nd	N/A	41.87d	1.44	115.39c	3.38	nd	N/A	14.67d	0.46	29.71d	1.34	36.72d	0.95	601.16a	19.26	279.24b	6.18
γ-Terpinene	nd	N/A	nd	N/A	8.79c	0.07	nd	N/A	nd	N/A	nd	N/A	7.82c	0.02	26.43a	0.69	15.75b	0.20
β-cis-Ocimene	36.92d	2.32	90.13d	2.91	227.08c	6.60	2.12d	0.26	44.46d	0.79	62.31d	2.62	75.26d	2.77	1176.79a	37.71	567.18b	11.97
Terpinolene	7.51d	0.03	8.71c	0.05	11.89c	0.14	nd	N/A	7.48c	0.02	7.98c	0.04	8.34c	0.03	41.82a	1.14	20.90b	0.29
cis Rose oxide	16.64d	0.01	16.68cd	0.01	16.95c	0.02	nd	N/A	16.44d	0.01	16.55d	0.01	16.68cd	0.01	22.74a	0.18	19.24b	0.04
trans-Rose oxide	16.41cd	0.00	16.43cd	0.00	16.52c	0.01	nd	N/A	16.35d	0.00	16.39d	0.00	16.42cd	0.00	18.50a	0.06	17.32b	0.01
(E,Z)-Allo-Ocimene	nd	N/A	12.13d	0.13	39.53c	1.34	nd	N/A	4.04d	0.18	8.00d	0.05	12.19d	0.21	202.00a	6.49	94.72b	1.98
Allo-Ocimene	nd	N/A	4.47cd	0.08	18.92c	0.73	nd	N/A	nd	N/A	2.36d	0.02	4.54cd	0.13	125.31a	4.10	53.04b	1.17
nerol oxide	65.11d	0.51	66.88d	0.59	96.93c	1.50	48.89d	0.37	53.89d	0.90	56.89d	0.23	100.79c	1.34	469.91a	14.54	218.50b	2.58
Citronellal	292.93d	0.92	372.55d	3.09	471.13bc	6.56	236.55d	1.31	306.75d	3.15	347.35d	4.49	365.23d	5.29	1230.16a	31.75	676.88b	7.85
Neral	14.64de	0.05	14.67de	0.03	18.75c	0.16	13.70e	0.02	14.18de	0.09	14.39de	0.02	16.33d	0.11	43.87a	0.73	24.21b	0.16
geranial	14.27e	0.03	14.71de	0.03	17.55c	0.12	13.78e	0.01	14.41e	0.08	14.73de	0.04	15.71d	0.04	30.87a	0.47	19.83b	0.09
cis-isogeraniol	nd	N/A	8.38cd	0.13	11.89bc	0.25	nd	N/A	nd	N/A	6.22cd	0.05	6.56cd	0.07	40.45a	1.15	18.46b	0.22
Geranic acid	579.99d	14.63	367.87d	21.20	2921.57b	109.64	185.55d	15.29	308.82d	28.46	450.35d	8.47	1775.95c	15.06	4971.88a	134.20	967.30cd	15.75
Linalool	22.12c	0.10	175.18a	5.19	179.63a	4.87	17.57c	0.06	27.94c	0.81	71.82b	1.96	28.53c	0.28	204.30a	6.24	102.95b	1.34
α-Terpineol	31.96c	0.03	34.06c	0.10	39.58b	0.31	30.85c	0.05	32.64c	0.20	33.07c	0.10	33.84c	0.15	66.02a	1.15	42.13b	0.23
Nerol	228.83c	3.79	506.78c	13.28	824.18c	21.56	86.67c	0.65	205.77c	2.90	308.27c	11.35	298.33c	7.02	4215.95a	129.51	2267.67b	37.62
Geraniol	146.64d	1.56	223.76d	4.15	442.51c	10.72	96.09d	0.61	181.65d	0.72	230.27d	6.39	257.38d	2.88	1130.06a	31.58	642.51b	8.88
Citronellol	12.48e	0.95	36.26d	0.98	64.26c	1.10	17.44de	0.48	14.32e	0.33	22.21de	0.16	32.69de	0.34	190.26a	4.70	116.94b	1.51
Limonene	7.20d	0.22	12.71cd	0.23	22.03c	0.41	8.35cd	0.11	7.62cd	0.08	9.45cd	0.04	12.55cd	0.08	112.07a	3.38	59.24b	1.08
α-muurolene	nd	N/A	nd	N/A	nd	N/A	nd	N/A	nd	N/A	nd	N/A	nd	N/A	nd	N/A	nd	N/A
cis-furan linalool oxide	nd	N/A	18.90b	0.03	18.24c	0.03	nd	N/A	nd	N/A	nd	N/A	nd	N/A	21.03a	0.02	18.38c	N/A
trans-furan linalool oxide	nd	N/A	nd	N/A	nd	N/A	nd	N/A	nd	N/A	nd	N/A	nd	N/A	nd	N/A	nd	N/A
Linalool oxide pyranoside	nd	N/A	nd	N/A	nd	N/A	nd	N/A	nd	N/A	nd	N/A	nd	N/A	19.77a	0.01	nd	N/A
trans-isogeraniol	nd	N/A	3.74cd	0.01	4.97bc	0.02	nd	N/A	nd	N/A	nd	N/A	nd	N/A	17.38a	0.52	7.97b	0.10

X, Y, Z represent the cultivars Xiangfei, Italia and Zaomeiguixiang. Different letters represent significant levels of P < 0.05, and nd stands for nondetected. N/A stands for not available, SE stands for standard error.
