# Supplementary material for: The accumulation profiles of terpene metabolites in three Muscat table grape cultivars through HS-SPME-GCMS
Source: Sci Data. 2020 Jan 2;7:5. doi: 10.1038/s41597-019-0321-1 (PMC6940363; doi:10.1038/s41597-019-0321-1)
Supplement: Supplementary file 1 [file 41597_2019_321_MOESM1_ESM.docx]

DATA-read.csv(file = DATA.CSV, header = TRUE)

mydata - ((DATA[,-1]))

rownames(mydata) - DATA$Samples

library(FactoMineR)

Result - PCA(mydata)

print(Result)

plot(Result)
